# Long-term seasonal dominance of the wasp *Trihapsis polita* Townes (Hymenoptera, Ichneumonidae) in the Brazilian Atlantic Forest

**DOI:** 10.3897/BDJ.5.e11337

**Published:** 2017-01-04

**Authors:** Bernardo F Santos, Alexandre P Aguiar, Anazélia M Tedesco, Julio C R Fontenelle

**Affiliations:** 1 Division of Invertebrate Zoology, American Museum of Natural History, New York, United States of America; 2 Departamento de Ciências Biológicas, Universidade Federal do Espírito Santo, Vitória, Brazil; 3 Secretaria de Estado de Meio Ambiente e Recursos Hídricos, Cariacica, Brazil; 4 Instituto Federal de Minas Gerais, Campus Ouro Preto, Ouro Preto, Brazil

**Keywords:** Malaise trap, parasitoid, phenology, outbreak, Trihapsis polita, secondary forest

## Abstract

**Background:**

The temporal dynamics of insect populations in tropical environments is highly complex and poorly known. Long-term seasonality studies are scarce, and particularly so for ichneumonid wasps (Hymenoptera Ichneumonidae). This study represents an effort to elucidate aspects of seasonality and forest succession in the Brazilian Atlantic Forest.

**New information:**

We report on the seasonal and successional dominance of the ichneumonid wasp *Trihapsis polita* (Cryptinae). A long-term survey of Cryptinae was carried out in a protected area of Brazilian Atlantic Forest, in primary, tall secondary and low secondary forest areas. Specimens were collected during rainy season (RS) and dry season (DS) between 2000 and 2008, with total sampling effort of 4,095 trap-days. A total of 8,385 specimens of Cryptinae were collected, of which 6,655 (79.4%) belonged to *T. polita*. The occurrence of *T. polita* species was heavily concentrated in the RS, with abundance 148× higher than during the DS. Seasonal fluctuation was also detected for Cryptinae as a whole, but was two orders of magnitude lower. Sampling efficiency also varied widely among areas, with the peak of abundance at the tall secondary forest. The dominance of *T. polita* in secondary vegetation might be of general interest, as this type of forest is currently on the rise, due to unprecedented levels of human pressure.

## Introduction

The temporal dynamics of insect populations in rainforests is highly complex and poorly studied (Hamer et al. 2005;Wolda 1988). Long-term studies of insect seasonality in the tropics are scarce, largely due the difficulty in maintaining long-term sampling and sorting (Grimbacher and Stork 2009). For Ichneumonidae, one of the largest insect families and a dominant component of tropical faunas, data are particularly scarce, deriving mostly from species that are easy to collect and identify (Gauld 2006). For Cryptinae, its largest and least studied subfamily, with 408 genera and over 4,500 species, there are no detailed studies of seasonality for any Neotropical taxon. Cryptines occur in all terrestrial environments and are mostly ectoparasitoids of pupae and prepupae of a wide variety of hosts, including moths, butterflies, beetles, social and solitary wasps.

**Figure 1.**
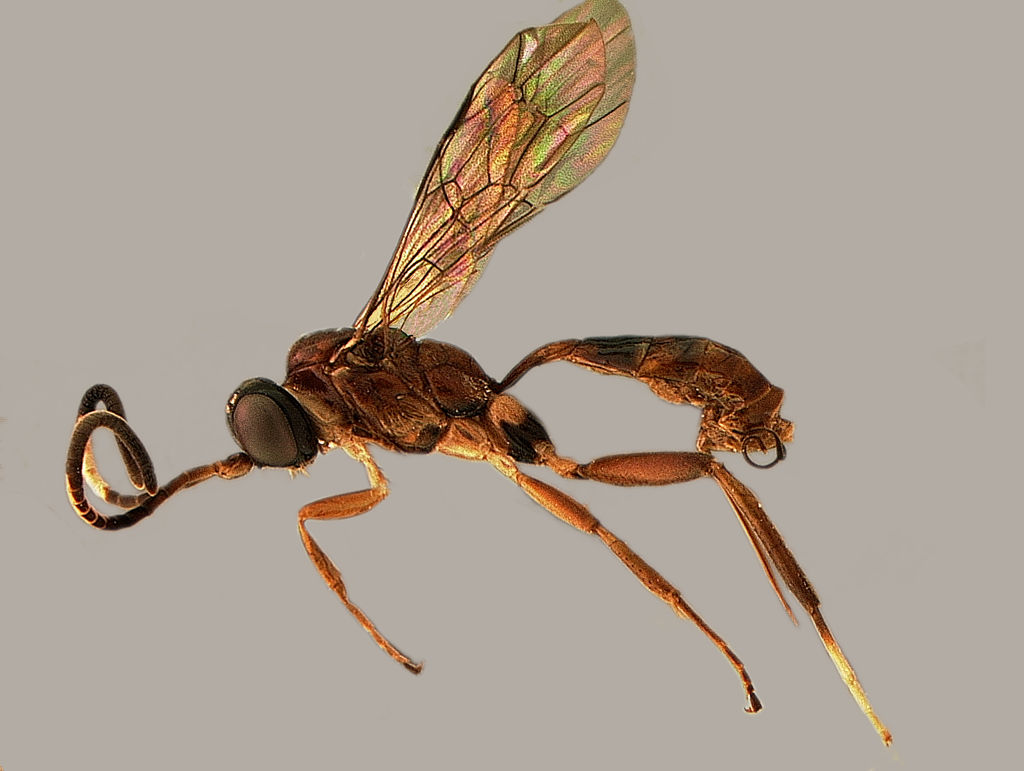
Female *Trihapsis polita* Townes, habitus.

Herein we report the seasonal distribution of a dominant cryptine species, *Trihapsis polita* Townes (Fig. 1). *Trihapsis* Townes is known from two Brazilian species, *T. punctata* Townes, and *T. polita* Townes, both described from a few specimens (Townes 1970). The genus seems to be restricted to the Brazilian Atlantic Forest, but is not frequently collected: a single specimen was obtained after an extensive collecting program in that region (Aguiar and Santos 2010). The hosts and other biological aspects of the genus are still unknown.

## Sampling methods

### Sampling description

Fieldwork was conducted over the course of eight years (2000–2008). In each year, one field excursion was conducted during the dry season (DS, July-August), and one during the rainy season (RS, October-November). Three Malaise traps (Townes 1972) were set in each site, aligned and 25 meters apart from each other. The traps were left operating for 4–5 weeks; the content of each trap was collected every 7 days and treated as a sample. Samples from the dry season of 2006 could not be accessed and were not considered.

Total sampling effort was 4,095 trap-days, 1,827 in the DS and 2,268 in the RS. Sampling efficiency is expressed as the ratio between number of specimens and number of trap-days. The number of collected specimens and sampling efficiency for *T. polita* were compared to that of all other Cryptinae combined in order to contextualize its relative abundance and to compare the temporal dynamics of this particular species to the “background” of closely related species in the area. All specimens are deposited in the entomological collection of *Universidade Federal do Espírito Santo* (Brazil). The full dataset is available as supplementary material (Suppl. material 1).

## Geographic coverage

### Description

**Figure 2.**
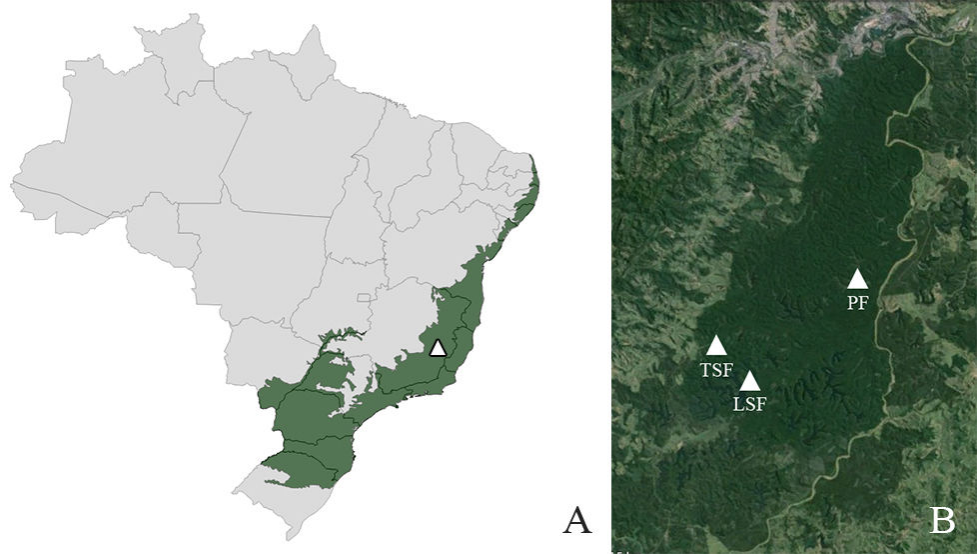
Map showing the study area. A, Brazilian Atlantic Forest (green) with the location of *Parque Estadual do Rio Doce* highlighted (white triangle). B, Map of the study area, with the three sampled sites highlighted. *PF*, primary forest; *TSF*, tall secondary forest; *LSF*, low secondary forest.

The study was conducted at *Parque Estadual do Rio Doce*, a 36,000 ha protected area in the Brazilian Atlantic Forest, state of Minas Gerais. Three sites within the park were sampled (Fig. 2): (1) an area of primary forest (PF) with well-developed canopy, overspread high trees, abundant bamboo trees and well developed understory; (2) tall secondary forest (TSF), an elevated, steep area; (3) an area of low secondary forest (LSF), dominated by bamboos.

### Coordinates

19˚48’18” and 19˚29’24” Latitude; 42˚38’’30” and 42˚28’’8” Longitude.

## Usage rights

### Use license

Creative Commons Public Domain Waiver (CC-Zero)

## Data resources

### Data package title

Raw_data

### Resource link

http://www.systaxon.ufes.br/pubs/Tpolita/

### Number of data sets

1

### Data set 1.

#### Data set name

Raw_data

#### Data format

Excel

#### Number of columns

9

#### Description

Original raw data: number of female and male specimens of *Trihapsis polita* and other Cryptinae in each collected sample.

**Data set 1.** 

Column label	Column description
Area	Sampled area by successional state. LSF, low secondary forest; HSF, high secondary forest; PF, primary forest.
Dates-Start	Starting day for that sample, corresponding to the day when the Malaise trap sample corresponding was initially set.
Dates-End	Ending day for that sample, corresponding to the day when the Malaise trap sample was collected.
Season	Categorical variable indicating whether sample was collected during the dry season (DR) or rainy season (RS).
Cryptinae	Number of specimens of Cryptinae (except T. polita) collected in that sample.
Trihapsis polita-Males	Number of male specimens of T. polita collected in that sample
Trihapsis polita-Females	Number of female specimens of T. polita collected in that sample
Total	Total number of specimens of T. polita collected in that sample
Pluviosity (mm)	Ammount of precipitation recorded during the week at which the sampling took place.

## Additional information

### Results and Discussion

**Figure 3.**
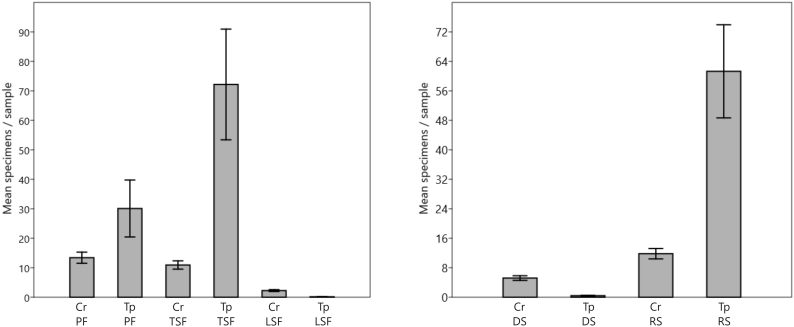
Mean number of specimens per sample (total of 3 traps; 195 samples/8 years), with standard error bars, comparing *T. polita* (Tp) with other Cryptinae (Cr) in three successional areas, primary forest (PF), tall secondary forest (TSF), and low secondary forest (LSF) (left chart), and in the dry season (DS) vs. rainy season (RS) (right chart). PERD.

**Figure 4.**
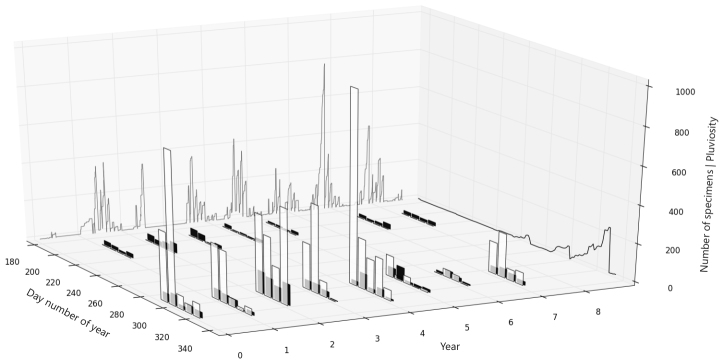
Total number of specimens collected in each sampling week (=each black & white bar pair, equivalent to 3 areas x 3 traps) for *T. polita* (white, first plane) vs. other Cryptinae (black, behind), along the sampled seasons (Day number of year, where DS=183–215, RS=286–322), discriminated for each year (0–1 = 2000–2008). Left wall (YZ axes) shows daily records of precipitation (cumulative values within each month), for all years (2893 records total); right wall (YX axes) shows average of precipitation for each Day number of year (each day = average of all years). Scale for number of specimens and precipitation (Y-axis) are identical.

A total of 8,384 specimens of Cryptinae were collected, of which 6,657 (79.4%) belonged to *T. polita*, and 1,727 (20.6%) to all other Cryptinae combined (Table 1). The overall sampling efficiency was 2.04. For *T. polita* alone, sampling efficiency was 1.62, and for all the other Cryptinae combined, 0.42. This quantitative superiority, however, was not uniform (Fig. 3). During the dry season (DS), *T. polita* represented only about 7% of the total Cryptinae collected. This situation was then drastically reversed in the rainy season (RS), when this ratio jumped to nearly 85%. The differences between dry and rainy seasons remain evident if the weekly totals are independently compared and are again clear if each week (Fig. 4) is compared separately, year by year.

For Cryptinae as a whole, sampling efficiency during the RS was 0.56, versus 0.25 in the DS, that is, 2.25× higher in the rainy season. The increase matches the observations of Kumagai (2002) for the Ichneumonidae in a preserved area in the Atlantic Forest, which tripled in number in the traps in the RS. This indicates that a two to threefold increase during the rainy season could be “normal” for Ichneumonidae as a whole. For *T. polita*, however, seasonal influence was higher by two orders of magnitude, with sampling efficiency rising from 0.02 in the DS to 2.92 in the RS – a 146× increase (Table 2).

The seasonal dominance of *T. polita* in the park was not a happenstance: it occurred repeatedly through the years (Fig. 4), as evidenced by its nearly uninterrupted numerical superiority in the samples (Table 3), and by its presence in 47.8% of the individual trap samples from the RS, against only 9.2% for the DS.

There was a distinctive quantitative difference among the three successional areas for the occurrence of both *T. polita* and other Cryptinae (Table 1). All differences discussed next are statistically significant (see confidence interval bars in Fig. 3). For other Cryptinae, the primary forest (PF) area had the highest abundance (871 specimens, 50.4% of the total), closely followed by the tall secondary forest (TSF) (710; 41.1%) and low secondary forest (LSF) (146; 8.5%). For *T. polita*, however, the TSF supported a much higher number of specimens (4,691; 70.5%), followed by the PF (1,956; 29.4%). The LSF was clearly avoided by this species, with only 10 specimens collected there throughout the study (0.15%).

Parasitoid wasps in general are most abundant in the rainy season, as compared to dry periods (Gauld 1991). However, this relationship remains insufficiently discussed and documented in the literature. The high level of dominance documented for *T. polita* has never been reported for any species of Ichneumonidae in natural areas, representing a noteworthy fact by itself. Previous faunistic studies of ichneumonid wasps in the Neotropics (e.g. Aguiar and Santos 2010; Guerra and Penteado-Dias 2002; Kumagai and Graf 2000; Kumagai and Graf 2000; Saaksjarvi et al. 2004) have never report any species as particularly dominating the collected samples. Considering that studies at the species level are scant for Ichneumonidae, the case for *T. polita* becomes of further interest, both because it is a rare study and because it signals that there is much still to be gained from studies on ichneumonid ecology.

The notorious success of *T. polita* in the tall secondary forest, as well as its distinct population dynamics in relation to other Cryptinae, suggest it may naturally act as a large-scale parasitoid in intermediate successional stages. This might also reveal to be of more general interest, now or in the future: this type of forest is predicted to increase in the 21st century due to industrialization and urbanization (Thomlinson et al. 1996), and yet, "*there is still a tremendous need to understand and further refine our knowledge of ecological processes involved in secondary succession*" (Guariguata and Ostertag 2001).

## Supplementary Material

Supplementary material 1 Raw data Data type: collecting records
Brief description: Original data, discriminated by individual trap-samples (585 records), presented as Python lists (compatible with R, and most other computer languages). All variables contain 585 items, with data corresponding according to position (index).
File: oo_111841.txt B.F.Santos, A.P.Aguiar & A.M.Tedesco

## Figures and Tables

**Table 1.** Total number of specimens collected for *T. polita* vs. other Cryptinae in all areas (*PF*, primary forest; *TSF*, tall secondary forest; *LSF*, low secondary forest) and seasons (*DS*, dry season; *RS*, rainy season). Sampling effort was identical for all sampling areas, adding to 4,113 trap-days total, but different between seasons (DS=1,836, RS=2,277).

	**Specimens**							%			
	**PF**	**TSF**	**LSF**	**Total**	**DS**	**RS**		**PF**	**TSF**	**LSF**	**Total**
*Trihapsis polita*	1956	4691	10	6657	36	6621		69.2	86.9	6.4	79.4
other Cryptinae	871	710	146	1727	416	1275		30.8	13.1	93.6	20.6
**Total**	2827	5401	156	8384	452	7896		33.7	64.4	1.9	100.0

**Table 2.** Comparison of sampling efficiency during the dry season (DS) vs. rainy season (RS) in the three sampled successional areas. *RS/DS*, how many times larger was the sampling efficiency in the rainy season in relation to the dry season. Sampling effort was identical for all areas, adding to 4,113 trap-days total, but different between seasons (DS=1,836, RS=2,277). %, percentage of specimens.

	***T. polita***					**other Cryptinae**			
**Area**	%	**DS**	**RS**	**RS/DS**		%	**DS**	**RS**	**RS/DS**
Primary forest (PF)	29.4	0.03	2.55	82.2		50.5	0.37	0.85	2.3
Tall secondary forest (TSF)	70.5	0.02	6.16	251.4		41.1	0.26	0.72	2.8
Low secondary forest (LSF)	0.2	0.00	0.01	3.2		8.4	0.10	0.11	1.1
PERD Total	79.4	0.02	2.91	148.3		20.6	0.25	0.56	2.3

**Table 3.** Number of females and males of *T. polita* collected, as opposed to all other Cryptinae combined. % ♀, the percentage of specimens of *T. polita* that are females; % *T. polita*, the percentage of the total specimens that correspond to *T. polita*

**Season, Year**	***T. polita*** ♀	***T. polita*** ♂	**other Cryptinae**	% ♀	% ***T. polita***
RS, 2000	695	648	173	51.75%	88.59
DS, 2001	0	3	64	0.0%	4.48%
RS, 2001	370	280	135	56.92%	82.80%
DS, 2002	6	6	129	50.00%	8.51%
RS, 2002	639	758	382	45.74%	78.53%
DS, 2003	3	6	82	33.33%	9.89%
RS, 2003	137	637	110	17.70%	87.56%
DS, 2004	2	3	34	40.00%	12.82%
RS, 2004	1084	606	150	64.14%	91.85%
DS, 2005	1	1	27	50.00%	6.90%
RS, 2005	131	49	142	72.78%	55.90%
RS, 2006	23	45	62	33.82%	52.31%
DS, 2007	1	1	60	50.00%	3.23%
RS, 2007	364	155	121	70.13%	81.09%
DS, 2008	1	0	59	100%	1.67%
Overall DS	14	20	455	41.18%	6.95%
Overall RS	3443	3178	1275	52.00%	83.85%
**Total**	**3457**	**3198**	**1730**	**51.95**%	**79.37**%
